# Cardiovascular Risk Assessment Among Adolescents and Youths Living With HIV: Evaluation of Electronic Health Record Findings and Implications

**DOI:** 10.2196/41574

**Published:** 2023-08-16

**Authors:** Sitaji Gurung, Kit N Simpson, Christian Grov, H Jonathon Rendina, Terry T K Huang, Henna Budhwani, Stephen Scott Jones, Tyra Dark, Sylvie Naar

**Affiliations:** 1 Department of Health Sciences New York City College of Technology (City Tech) The City University of New York Brooklyn, NY United States; 2 Department of Healthcare Leadership and Management Medical University of South Carolina Charleston, SC United States; 3 Department of Community Health and Health Policy The City University of New York Graduate School of Public Health and Health Policy New York, NY United States; 4 Milken Institute School of Public Health The George Washington University Washington, DC United States; 5 Center for Systems and Community Design The City University of New York Graduate School of Public Health and Health Policy New York, NY United States; 6 College of Nursing Florida State University Tallahassee, FL United States; 7 Whitman-Walker Institute The George Washington University Washington, DC United States; 8 College of Medicine Florida State University Tallahassee, FL United States

**Keywords:** cardiovascular risk, cluster of differentiation 4 lymphocyte, electronic health record, viral load, youths living with HIV

## Abstract

**Background:**

The HIV epidemic remains a major public health concern, particularly among youths living with HIV. While the availability of antiretroviral therapy has significantly improved the health outcomes of people living with HIV, there is growing evidence that youths living with HIV may be at increased risk of cardiovascular disease. However, the underlying mechanisms linking HIV and cardiovascular disease among youths living with HIV remain poorly understood. One potential explanation is that HIV-related biomarkers, including detectable viral load (VL) and low cluster of differentiation 4 (CD4) lymphocyte counts, may contribute to increased cardiovascular risk. Despite the potential importance of these biomarkers, the relationship between HIV-related biomarkers and cardiovascular risk among youths living with HIV has been understudied.

**Objective:**

To address this gap, we examined whether detectable VL and low CD4 lymphocyte counts, both of which are indications of unsuppressed HIV, were associated with cardiovascular risk among youths living with HIV.

**Methods:**

We analyzed electronic health record data from 7 adolescent HIV clinics in the United States (813 youths living with HIV). We used multivariable linear regression to examine the relationship between detectable VL and CD4 lymphocyte counts of ≤200 and cardiovascular risk scores, which were adapted from the gender-specific Framingham algorithm.

**Results:**

In our study, nearly half of the participants (366/766, 47.8%) had detectable VL, indicating unsuppressed HIV, while 8.6% (51/593) of them had CD4 lymphocyte counts of ≤200, suggesting weakened immune function. We found that those with CD4 lymphocyte counts of ≤200 had significantly higher cardiovascular risk, as assessed by Cardiac Risk Score2, than those with CD4 lymphocyte counts of >200 (*P*=.002). After adjusting for demographic and clinical factors, we found that for every 1000-point increase in VL copies/mL, the probability of having cardiovascular risk (Cardiac Risk Score2) increased by 38%. When measuring the strength of this connection, we observed a minor effect of VL on increased cardiovascular risk (β=.134, SE 0.014; *P*=.006). We obtained similar results with Cardiac Risk Score1, but the effect of CD4 lymphocyte counts of ≤200 was no longer significant. Overall, our findings suggest that detectable VL is associated with increased cardiovascular risk among youths living with HIV, and that CD4 lymphocyte counts may play a role in this relationship as well.

**Conclusions:**

Our study highlights a significant association between unsuppressed HIV, indicated by detectable VL, and increased cardiovascular risk in youths living with HIV. These findings emphasize the importance of implementing interventions that address both VL suppression and cardiovascular risk reduction in this population. By tailoring interventions to meet the unique needs of youths, we can promote overall well-being throughout the HIV care continuum and across the life span. Ultimately, these efforts have the potential to improve the health outcomes and quality of life of youths living with HIV.

**International Registered Report Identifier (IRRID):**

RR2-10.2196/11185

## Introduction

Youths living with HIV is a high-priority population within those living with HIV [1]. Youths living with HIV will need to manage their diagnosis for years longer than their adult peers, and negative health consequences associated with living with HIV may appear earlier in the life course [2-6]. Research shows significant gaps in the HIV treatment cascade in younger populations compared to older groups [7-9]. Youths living with HIV aged 18-24 years have lower uptake of HIV testing and lower rates of treatment initiation compared to older peers, which continues to present a significant challenge to epidemic control [8-10]. Notably, racial disparities are more pronounced in youths [11,12]. Youths who identify as Black and Latinx represent 54% and 25% of new HIV diagnoses (79% in aggregate), compared to those who identify as White and other races, accounting for 16% and 5% of new HIV diagnoses [13]. There is growing evidence to suggest that Black individuals are at a higher risk for cardiovascular disease (CVD) compared to individuals in other racial and ethnic groups [14]. Several factors contribute to this increased risk. One of the most significant is systemic racism and discrimination, which can result in chronic stress and inflammation that can damage the heart and blood vessels over time [15]. According to the American Heart Association, Black individuals are more likely to have hypertension and less likely to have it under control compared to other racial and ethnic groups [16]. Black individuals are also more likely to develop diabetes and are at a higher risk of dying from diabetes-related complications. These conditions are major risk factors for CVD and contribute to the increased risk seen in Black individuals.

Cardiovascular health disparities are well documented among older adults with HIV [17-22], but there is a dearth of research on cardiovascular health metrics and prevention strategies for youths living with HIV in the United States. Studies have shown that older adults with HIV have a higher risk of developing CVD compared to the general population, which is likely due to a combination of factors, including HIV-related inflammation, the use of antiretroviral therapy (ART), and lifestyle factors, such as smoking and poor diet [21,23,24]. However, there is a lack of research on the cardiovascular health of youths living with HIV in the United States, despite the fact that this population is growing and faces unique challenges to their health. Research on cardiovascular health metrics is therefore critical to the overall understanding of the impact of HIV infection on behavioral factors and the relationship between HIV infection and cardiovascular risk. Research has suggested that indicators of CVD may appear earlier in individuals living with HIV, with some studies suggesting that these indicators can emerge as early as adolescence [4,6,25], potentially due to a combination of factors. One factor is that HIV infection can cause chronic inflammation, which has been linked to the development of atherosclerosis, a key contributor to CVD [26,27]. Another factor is that some ARTs used to manage HIV infection may have adverse effects on lipid metabolism, leading to the accumulation of fatty deposits in the blood vessels and an increased risk of CVD [28].

In addition to these factors, individuals living with HIV may also have a higher prevalence of traditional CVD risk factors, such as hypertension, diabetes, and smoking, which can further increase their risk of developing CVD [29]. Stigma and discrimination related to HIV infection can also contribute to poor mental health outcomes, which have been linked to an increased risk of CVD [30]. Given the potential for CVD indicators to emerge earlier in individuals living with HIV, it is important to develop effective prevention and treatment strategies targeted toward this population. This may include identifying and managing traditional CVD risk factors, optimizing ART to minimize potential negative effects on lipid metabolism, and addressing mental health concerns through counseling and other interventions. The American Heart Association’s Life’s Simple 7 (LS7) concept of ideal cardiovascular health is not well understood in youths living with HIV when compared to the Healthy People 2020-2030 goals [31-34]. A higher LS7 score indicates better cardiovascular health and is associated with a lower incidence of CVD [35-38]. Traditional assessments of individual cardiovascular risk factors are inadequate in capturing the overall risk posed by multiple factors at the population level [39]. It is important to consider risk factors that occur together [40,41] as cardiovascular risk factor profiles [42-44] and assess them using composite measures such as the Framingham Risk Score (FRS) [45]. The modified FRS is the most commonly used algorithm for cardiovascular risk assessment in the United States [45,46].

Reducing cardiovascular risk in youths living with HIV is a critical issue that requires attention. It is essential to conduct cardiovascular risk assessment in this population to optimize the early diagnosis and treatment of CVD during adolescence [47]. However, research focused on comorbidities in HIV has rarely explored the connection between detectable viral load (VL) and low cluster of differentiation 4 (CD4) lymphocyte counts with increased cardiovascular risk in a US-based population of youths living with HIV. Additionally, there are currently no published data on the prevalence of traditional cardiovascular risk factors, such as dyslipidemia, hypertension, diabetes, and smoking, among youths living with HIV aged 14-26 years, even though metabolic changes leading to atherosclerosis can begin early in life, and go undiagnosed for an extended period. Despite this, cardiac risk estimating algorithms like the FRS have not been applied to younger populations [48,49]. Therefore, the aim of this study was to develop cardiovascular risk profiles for a cohort of US-based youths living with HIV and compare their profiles based on VL and CD4 lymphocyte status.

## Methods

### Study Design and Population

The study analyzed deidentified electronic health record (EHR) data of adolescents for the Adolescent Medicine Trials Network protocols. The Adolescent Medicine Trials Network protocols were previously described in a publication [50], and the participating sites included in the EHR extraction protocol [51] provide HIV care to youths living with HIV. This care begins with a diagnosis, followed by linking them to an HIV care provider who can help manage their HIV on a regular basis.

### Ethics Approval

The institutional review board (IRB) of Florida State University granted approval (STUDY00000549) for the analysis of deidentified data, which was carried out in compliance with the US Department of Health and Human Services 45 Code of Federal Regulations Part 46. The parent protocol did not involve an informed consent process, and a waiver of consent and HIPAA (Health Insurance Portability and Accountability Act) waiver were granted. To protect confidentiality, the data were deidentified and participants were identified only by a unique study ID. The data were analyzed in aggregate to compare the clinic site with individuals, and a centralized data extraction process was used at all sites. Each participating site received a signed statement confirming the IRB’s approval before the study began, and a reliance agreement was obtained from each site indicating their commitment to providing deidentified EHR data in accordance with the IRB’s approval.

The extracted EHR data included those who had standard of care and treatment visits at one of the participating sites between January 1, 2016, and December 31, 2016. The baseline data extraction cycle was launched in December 2017 and completed in April 2018. Once the data files were verified for each site, they were merged to produce a single analytic data set of 1093 youths living with HIV. To be included in these analyses, data on demographics, HIV biomarkers, vital statistics, cholesterol panel, and the International Statistical Classification of Disease and Related Health Problems, Tenth Revision (ICD-10), diagnosis codes were required. Data downloads from Baltimore, Maryland; Birmingham, Alabama; Los Angeles, California; Memphis, Tennessee; San Diego, California; Tampa, Florida; and Washington, District of Columbia; were included in the analyses. Because Brooklyn, New York; Miami, Florida; and Philadelphia, Pennsylvania; did not provide gender and systolic blood pressure data, these 3 sites were excluded from further analyses.

### Measures

#### Demographics

We defined age as a continuous variable in years as reported by the site. Gender, race, and ethnicity definitions varied within a site’s data download. For gender, we constructed a binary variable of man and woman. Race and ethnicity were categorized into Black, Latinx, White, and other.

#### HIV and Immunologic Biomarkers

Earliest VL values in “copies/mL” by date were used from each site. VL was log-transformed for analysis as a continuous outcome. The detectable VL threshold as reported by the site was coded as a dichotomous variable. Based on the guidelines for the use of antiretroviral agents in HIV-1 infected adults and adolescents, we defined immunocompromised as a CD4 T lymphocyte count of less than or equal to 200 [52,53]. CD4 lymphocyte count was divided by 100 to enhance the interpretability of the coefficients to correspond to a 100-cell increase rather than a 1-cell increase.

#### ART Medication

All prescribed HIV medications were coded into a dichotomous variable, where 0=no prescribed medication and 1=at least one prescribed medication.

#### Substance Abuse Diagnosis

We used ICD-10 codes F1010, F1210, F1220, F1290, F1510, F1511, F1520, F1590, F17200, F17210, F1910, and F1920 to identify alcohol and drug dependence. Substance abuse was treated as a dichotomous variable.

#### BMI

BMI was calculated based on weight and height (kg/m^2^). If multiple values for a given patient were present in EHR, values from the earliest date were used.

#### Cardiac Risk Scores

We adopted the gender-specific cardiovascular risk algorithm developed by the Framingham Heart Study [43] to construct a cardiovascular risk variable referred to as Cardiac Risk Score1 using clinic-based predictors that are routinely obtained in primary care and do not require laboratory testing. The variables required for constructing the Cardiac Risk Score1 include age, gender, systolic blood pressure, use of antihypertensive medication, smoking status, and diabetes status. As gender and systolic blood pressure data were required for the development of the Cardiac Risk Score1, those without these data were excluded from the first analytic sample*.*

**Figure 1:**
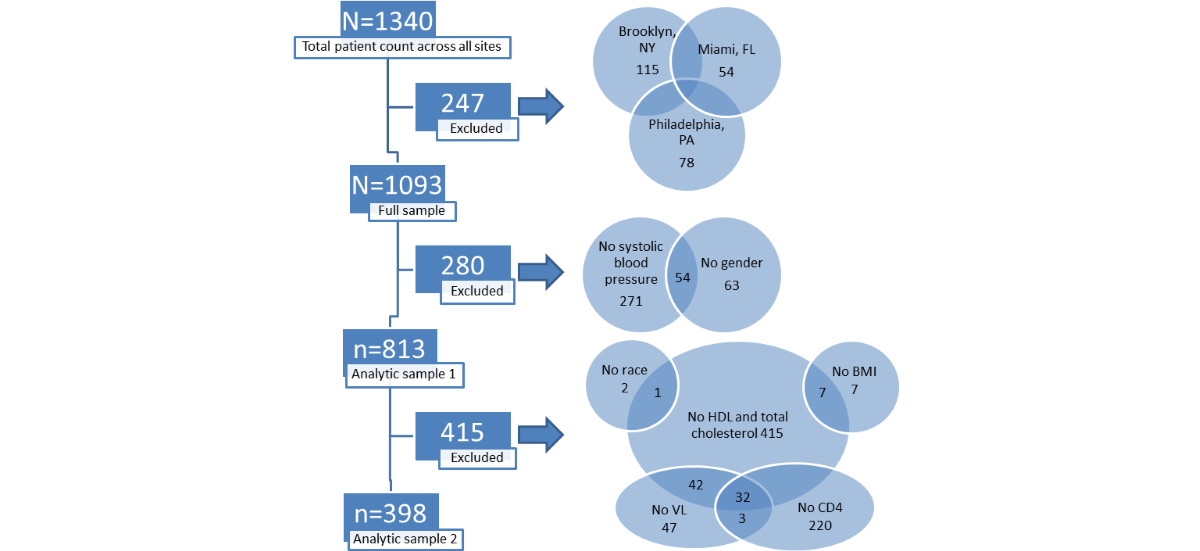
CONSORT (Consolidated Standards of Reporting Trials)-style diagram illustrating a comprehensive assessment of all missingness in the construction of Cardiac Risk Score1 and Cardiac Risk Score2. CD4: cluster of differentiation 4; HDL: high-density lipoprotein; VL: viral load.

We also constructed a second cardiovascular risk variable referred to as Cardiac Risk Score2 that used routinely obtained clinic-based predictors including laboratory testing. The variables required for constructing the Cardiac Risk Score2 include age, gender, systolic blood pressure, antihypertensive medication use, current smoking, diabetes, total cholesterol, and high-density-lipoprotein (HDL) cholesterol. As gender, total cholesterol, HDL cholesterol, and systolic blood pressure data were required for the development of the Cardiac Risk Score2, those without this data were excluded from the second analytic sample. The CONSORT (Consolidated Standards of Reporting Trials)-style diagram illustrates a comprehensive assessment of all missingness in the construction of Cardiac Risk Score1 and Cardiac Risk Score2 (Figure 1). We assigned an age value of 21 years. Following the convention of the existing CVD prediction algorithm developed based on data obtained from the Framingham Heart Study, we constructed a separate gender-specific multivariable risk function algorithm for men versus women [43]. The syntax for both Cardiac Risk Score1 and Cardiac Risk Score2 can be found in the Multimedia Appendix 1.

### Data Analysis

We included youths living with HIV who had health records of systolic blood pressure, antihypertensive medication use, current smoking, and diabetes status in 2016, in the first analytic sample for Cardiac Risk Score1 (n=813). Subsequently, we constructed a second analytic sample for Cardiac Risk Score2 (n= 398) by including total cholesterol and HDL cholesterol in addition to the clinic-based predictors mentioned above. As not all patients had records for these additional variables, the sample size of Cardiac Risk Score2 was reduced to 398.

We performed a descriptive analysis of the demographic and clinical characteristics of the sample, followed by bivariate analyses using the chi-square test and independent samples *t* test with detectable VL and low CD4 lymphocyte count as a dichotomous variable. Then, we ran 3 multivariable linear regression models with Cardiac Risk Score1 as a continuous outcome variable to determine the association between detectable VL and cardiovascular risk, impaired immune function (CD4 lymphocyte count ≤200) and cardiovascular risk, and the combined effect of detectable VL and low CD4 lymphocyte count with cardiovascular risk. We repeated these regression models with Cardiac Risk Score2 as the outcome variable. Covariates included age, race and ethnicity, gender, being prescribed ART medication, substance dependence, and BMI.

The linear regression coefficients were evaluated using unstandardized beta (B) as the point estimate, CI was 95% for the variability around that point estimate, and *P* value for statistical significance. Standardized beta (β) was used to estimate the effect size of independent variables on the dependent variable, categorized as small (.10), medium (.30), and large (.50) according to Cohen’s criteria. SPSS Statistics (version 25; IBM Corp) was used for all analyses.

## Results

### Overview

Table 1 provides a summary of the demographic and clinical characteristics of the analytic sample of the study, which includes 813 youths living with HIV. The mean age of the sample was 21 (SD 2.6) years. The sample was predominantly of racial and ethnic minorities, with 70.3% (570/811) of them identifying as Black and 10.9% (88/811) of them as Latinx. Additionally, the majority of the sample were men (563/813, 69.2%). The study also presents the prevalence of detectable VL and impaired immune health, indicating that 47.8% (366/766) of those with VL data had detectable VL and 8.6% (51/593) of those with CD4 lymphocyte data had a baseline count ≤200. The proportion of youths living with HIV who were currently taking ART was high at 88.6% (720/813). The mean BMI of the sample was 25 (SD 6.7) kg/m^2^, and the mean Cardiac Risk Scores 1 and 2 were 0.062 (SD 0.039) and 0.672 (SD 0.401), respectively.

**Table 1:** Demographic and clinical characteristics of the sample (N=813).

Characteristics			Values
**Race and ethnicity (n=811)**			
	Black	570 (70.3)	
	Latinx	88 (10.9)	
	White	81 (10)	
	Other	72 (8.9)	
**Gender (N=813)**			
	Man	563 (69.2)	
	Woman	250 (30.8)	
**ART^a^ medication (N=813)**			
	Currently on ART medication	720 (88.6)	
	Not reportedly on ART medication	93 (11.4)	
**VL^b^ (n=766)**			
	Detectable VL	366 (47.8)	
	Undetectable VL	400 (52.2)	
**CD4^c^ lymphocyte count (n=593)**			
	Baseline count ≤200	51 (8.6)	
	Baseline count >200	542 (91.4)	
**Substance abuse diagnosis (N=813)**			
	Diagnosed with substance abuse	99 (12.2)	
	Not diagnosed with substance abuse	714 (87.8)	
**ATN^d^ clinical site (N=813)**			
	Baltimore, Maryland	90 (11.1)	
	Birmingham, Alabama	61 (7.5)	
	Los Angeles, California	84 (10.3)	
	Memphis, Tennessee	187 (23)	
	San Diego, California	101 (12.4)	
	Tampa, Florida	219 (26.9)	
	Washington, District of Columbia	71 (8.7)	
Age (years), mean (SD); median (range)			21 (2.6); 21 (14-26)
BMI (kg/m^2^; N=806), mean (SD); median (range)			25 (6.7); 23.3 (4.8-69.5)
Cardiac Risk Score1^e^, mean (SD); median (range)			0.062 (0.040); 0.061 (0.01-0.26)
Cardiac Risk Score2^f^ (n=398), mean (SD); median (range)			0.672 (0.401); 0.569 (0.17-4.05)

^a^ART: antiretroviral therapy.

^b^VL: viral load.

^c^CD4: cluster of differentiation 4.

^d^ATN: Adolescent Medicine Trials Network.

^e^Cardiac Risk Score1: defined as the risk score for patients with systolic blood pressure, smoking, diabetes, and antihypertensive medication use.

^f^Cardiac Risk Score2: defined as the risk score for patients with systolic blood pressure, smoking, diabetes, antihypertensive medication use, total cholesterol, and high-density-lipoprotein cholesterol.

### Bivariate Correlates of HIV and Immunologic Biomarkers

#### Demographic and Clinical Correlates

Table 2 presents findings on the demographic and clinical correlates of the study population; the table highlights several general trends observed in the data. First, patients who had detectable VL were more likely to be Black than those who had undetectable VL. The proportion of Black patients was 74% (271/366) among those with detectable VL, compared to 67% (268/400) among those with undetectable VL. This difference was statistically significant (*χ*²_3_=9.9; *P*<.05). Second, patients with detectable VL were more likely to be men than those without detectable VL. Specifically, 72.7% (266/366) of patients with detectable VL were men, compared to 65.2% (261/400) of patients without detectable VL. This difference was also statistically significant (*χ*²_1_=4.9; *P*=.03). Finally, patients with detectable VL were less likely to be on ART medication than those with undetectable VL. Specifically, 87.7% (321/366) of those with detectable VL were on ART medication, compared to 92.5% (370/400) of those with undetectable VL. This difference was statistically significant (*χ*²_1_=5.0; *P*=.03). These findings suggest that demographic and clinical factors are important correlates of VL in this population.

The 10-year risk of developing CVD was estimated using 2 cardiovascular risk assessment variables, Cardiac Risk Score1 and Cardiac Risk Score2. In bivariate analyses, we found significant associations between VL and cardiovascular risk as measured by both cardiac risk scores. Specifically, the results show that the association between VL and cardiovascular risk was statistically significant for Cardiac Risk Score1 (*P*=.001) and Cardiac Risk Score2 (*P*=.007). However, no significant differences were found between the demographic characteristics and the immunologic biomarker, CD4 lymphocyte count, as displayed in Table 3. When comparing patients who had a CD4 lymphocyte count of ≤200 with those who had a CD4 lymphocyte count of >200, no significant difference was found in Cardiac Risk Score1. However, the results show that with Cardiac Risk Score2, the risk of CVD was significantly increased in patients who had a CD4 lymphocyte count of ≤200 compared with those who had a CD4 lymphocyte count of >200 (*P*=.002). These findings suggest that VL is associated with an increased risk of CVD as measured by both Cardiac Risk Score1 and Cardiac Risk Score2*,* and that CD4 lymphocyte count may be an important factor in predicting cardiovascular risk using Cardiac Risk Score2.

**Table 2:** Bivariate analysis by detectable viral load (VL; N=766).

Characteristics			Total sample		Detectable VL (n=366)		Undetectable VL (n=400)		*F* test (*df*)			Chi-square (*df*)			*P* value	
**Race and ethnicity (n=764), n (%)**										N/A^a^			9.881 (3)			.02
	Black	539 (70.5)		271 (74.5)		268 (67)										
	Latinx	85 (11.1)		43 (11.8)		42 (10.5)										
	White	73 (9.6)		27 (7.4)		46 (11.5)										
	Other	67 (8.8)		23 (6.3)		44 (11)										
**Gender (N=766), n (%)**										N/A			4.912 (1)			.03
	Man	527 (68.8)		266 (72.7)		261 (65.2)										
	Woman	239 (31.2)		100 (27.3)		139 (34.8)										
**ART^b^ medication (N=766), n (%)**										N/A			4.975 (1)			.03
	Currently on ART medication	691 (90.2)		321 (87.7)		370 (92.5)										
	Not reportedly on ART medication	75 (9.8)		45 (12.3)		30 (7.5)										
**CD4^c^ lymphocyte count (n=593), n (%)**										N/A			32.326 (1)			<.001
	Baseline count ≤ 200	50 (8.6)		42 (15.8)		8 (2.5)										
	Baseline count > 200	532 (91.4)		224 (84.2)		308 (97.5)										
**Substance abuse diagnosis (N=766),** **n (%)**										N/A			0.333 (1)			.56
	Diagnosed with substance abuse	97 (12.7)		49 (13.4)		48 (12)										
	Not diagnosed with substance abuse	669 (87.3)		317 (86.6)		352 (88)										
Age (years), mean (SD)			21 (2.6)		21 (2.5)		21 (2.7)		2.990 (764)			N/A			.58	
BMI (kg/m^2^; n=762), mean (SD)			25 (6.8)		25 (6.6)		26 (6.9)		3.626 (760)			N/A			.13	
Cardiac Risk Score1^d^, mean (SD)			0.062 (0.040)		0.067 (0.042)		0.058 (0.037)		2.049 (764)			N/A			.001	
Cardiac Risk Score2^e^ (n=394), mean (SD)			0.672 (0.672)		0.735 (0.452)		0.626 (0.348)		4.577 (392)			N/A			.007	

^a^N/A: not applicable.

^b^ART: antiretroviral therapy.

^c^CD4: cluster of differentiation 4.

^d^Cardiac Risk Score1: defined as the risk score for patients with systolic blood pressure, smoking, diabetes, and antihypertensive medication use.

^e^Cardiac Risk Score2: defined as the risk score for patients with systolic blood pressure, smoking, diabetes, antihypertensive medication use, total cholesterol, and high-density-lipoprotein cholesterol.

**Table 3:** Bivariate analysis by low cluster of differentiation 4 (CD4) lymphocyte count (N=593).

Characteristics			Total sample		CD4 lymphocyte count≤200 (n=51)		CD4 lymphocyte count>200 (n=542)		*F* test (*df*)			Chi-square (*df*)			*P* value	
**Race and ethnicity (n=591), n (%)**										N/A^a^			6.548 (3)			.09
	Black	431 (72.9)		44 (88)		387 (71.5)										
	Latinx	63 (10.7)		3 (6)		60 (11.1)										
	White	48 (8.1)		1 (2)		47 (8.7)										
	Other	49 (8.3)		2 (4)		47 (8.7)										
**Gender (N=593), n (%)**										N/A			0.002 (1)			.97
	Man	420 (70.8)		36 (70.6)		384 (70.8)										
	Woman	173 (29.2)		15 (29.4)		158 (29.2)										
**ART^b^ medication (N=593), n (%)**										N/A			1.397 (1)			.24
	Currently on ART medication	529 (89.2)		48 (94.1)		481 (88.7)										
	Not reportedly on ART medication	64 (10.8)		3 (5.9)		61 (11.3)										
**VL^c^ (n=582), n (%)**										N/A			32.326 (1)			<.001
	Detectable VL	266 (45.7)		42 (84)		224 (42.1)										
	Undetectable VL	316 (54.3)		224 (42.1)		308 (57.9)										
**Substance abuse diagnosis (N=593), n (%)**										N/A			0.445 (1)			.51
	Diagnosed with substance abuse	86 (14.5)		9 (17.6)		77 (14.2)										
	Not diagnosed with substance abuse	507 (85.5)		42 (82.4)		465 (85.8)										
Age (years), mean (SD)			21 (2.6)		21 (2.5)		21 (2.5)		0.042 (591)			N/A			.63	
BMI (kg/m^2^; n=591), mean (SD)			25 (6.8)		24 (5.8)		25 (7)		3.357 (589)			N/A			.09	
Cardiac Risk Score1^d^, mean (SD)			0.062 (0.039)		0.066 (0.046)		0.060 (0.037)		1.223 (591)			N/A			.35	
Cardiac Risk Score2^e^ (n=343), mean (SD)			0.672 (0.401)		0.864 (0.741)		0.621 (0.338)		8.712 (341)			N/A			.002	

^a^N/A: not applicable.

^b^ART: antiretroviral therapy.

^c^VL: viral load.

^d^Cardiac Risk Score1: defined as the risk score for patients with systolic blood pressure, smoking, diabetes, and antihypertensive medication use.

^e^Cardiac Risk Score2: defined as the risk score for patients with systolic blood pressure, smoking, diabetes, antihypertensive medication use, total cholesterol, and high-density-lipoprotein cholesterol.

#### Multivariable Analyses of Cardiac Risk Scores

The multivariable regression analysis of Cardiac Risk Score1 and Cardiac Risk Score2 scores is presented in Tables 4 and 5. For Cardiac Risk Score1, Model 1A showed a small effect of VL on increased cardiovascular risk (β=.067, SE 0.001; *P*=.008), while no significant association was found for CD4 lymphocyte count (Model 1B)*.* Model 1C also demonstrated a significant positive association between VL and cardiovascular risk (*P*=.007), but no significant association was found for CD4 lymphocyte count. The findings of Model 1A and Model 1C were consistent.

For Cardiac Risk Score2, Model 2A showed a 38% increase in the likelihood of having cardiovascular risk for a 1000-point increase in VL (B=.038, 95% CI 0.011-0.066). Model 2B showed no significant association between CD4 lymphocyte count and cardiovascular risk. Model 2C demonstrated a significant positive association between VL and cardiovascular risk (*P*=.01), while that with the CD4 lymphocyte count was not significant (*P*=.54). The findings of Model 2A and Model 2C were consistent. The standardized beta value for the effect of VL on cardiovascular risk was only over .1 in both Cardiac Risk Score1 and Cardiac Risk Score2, indicating a small effect size.

**Table 4:** Multivariable linear regression Model 1 by Cardiac Risk Scores. Cardiac Risk Score1: defined as the risk score for patients with systolic blood pressure, smoking, diabetes, and antihypertensive medication use.

Multivariable	Model 1A with VL^a^ findings							Model 1B with CD4^b^ lymphocyte findings							Model 1C with VL and CD4 lymphocyte findings					
	Unstandardized				Standardized			Unstandardized				Standardized			Unstandardized				Standardized	
	B	SE	95% CI	β		*P* value	B		SE	95% CI	β		*P* value	B		SE	95% CI	β		*P* value
Age	.001	0.000	0.000 to 0.002	.072		.005	.002		0.000	0.001 to 0.003	.123		<.001	.002		0.000	0.001 to 0.003	.135		<.001
Race and ethnicity	.002	0.001	0.000 to 0.004	.056		.03	.002		0.001	0.000 to 0.004	.043		.12	.001		0.001	–0.001 to 0.004	.036		.22
Gender	.061	0.002	0.057 to 0.065	.710		<.001	.058		0.002	0.053 to 0.063	.691		<.001	.059		0.003	0.054 to 0.064	.696		<.001
ART^c^ medication	.001	0.003	–0.005 to 0.008	.010		.70	–.001		0.003	–0.008 to 0.006	–.007		.81	.001		0.004	–0.006 to 0.008	.006		.83
Substance abuse diagnosis	.015	0.003	0.009 to 0.020	.124		<.001	.017		0.003	0.011 to 0.023	.159		<.001	.015		0.003	0.008 to 0.021	.136		<.001
BMI	.000	0.000	0.000 to 0.001	.057		.03	.000		0.000	0.000 to 0.001	.055		.06	.000		0.000	0.000 to 0.001	.053		.08
VL	.002	0.001	0.000 to 0.003	.067		.008	N/A^d^		N/A	N/A	N/A		N/A	.002		0.001	0.001 to 0.004	.088		.007
CD4 lymphocyte count	N/A	N/A	N/A	N/A		N/A	.000		0.000	–0.001 to 0.000	–.028		.32	.000		0.000	–0.001 to 0.001	.015		.65

^a^VL: viral load.

^b^CD4: cluster of differentiation 4.

^c^ART: antiretroviral therapy.

^d^N/A: not applicable.

**Table 5:** Multivariable linear regression Model 2 by Cardiac Risk Scores. Cardiac Risk Score2: defined as the risk score for patients with systolic blood pressure, smoking, diabetes, antihypertensive medication use, total cholesterol, and high-density-lipoprotein cholesterol.

Multivariable	Model 2A with VL^a^ findings								Model 2B with CD4^b^ lymphocyte findings								Model 2C with VL and CD4 lymphocyte findings					
	Unstandardized				Standardized			Unstandardized					Standardized			Unstandardized					Standardized	
	B	SE	95% CI	β		*P* value	B			SE	95% CI	β		*P* value	B			SE	95% CI	β		*P* value
Age	.012	0.008	–0.003 to 0.027	.081		.10	.023			0.008	0.007 to 0.039	.152		.004	.024			0.008	0.008 to 0.040	.158		.003
Race and ethnicity	.039	0.021	–0.002 to 0.081	.091		.06	.040			0.022	–0.002 to 0.082	.096		.06	.045			0.022	0.002 to 0.088	.105		.04
Gender	.164	0.044	0.077 to 0.251	.188		<.001	.163			0.045	0.074 to 0.252	.194		<.001	.170			0.046	0.080 to 0.259	.201		<.001
ART^c^ medication	–.030	0.073	–0.174 to 0.114	–.020		.68	–.080			0.070	–0.218 to 0.057	–.059		.25	–.069			0.071	–0.209 to 0.070	–.050		.33
Substance abuse diagnosis	.059	0.055	–0.049 to 0.166	.053		.29	.074			0.054	–0.032 to 0.180	.071		.17	.055			0.054	–0.053 to 0.162	.053		.32
BMI	.014	0.003	0.008 to 0.019	.245		<.001	.013			0.003	0.007 to 0.018	.236		<.001	.013			0.003	0.007 to 0.018	.240		<.001
VL	.038	0.014	0.011 to 0.066	.134		.006	N/A^d^			N/A	N/A	N/A		N/A	.040			0.016	0.009 to 0.071	.146		.01
CD4 lymphocyte count	N/A	N/A	N/A	N/A		N/A	–.012			0.007	–0.025 to 0.001	–.095		.07	–.005			0.007	–0.019 to 0.010	–.035		.54

^a^VL: viral load.

^b^CD4: cluster of differentiation 4.

^c^ART: antiretroviral therapy.

^d^N/A: Not applicable.

## Discussion

This study was among the first to examine the creation of cardiovascular risk profiles and assess their connections with HIV biomarkers in youths living with HIV aged 14-26 years. The results showed that detectable VL was statistically significantly linked to cardiovascular risk, even after adjusting for demographic and clinical factors, underscoring the need for integrating cardiovascular health and HIV care in regular clinical practice. The study also revealed that higher plasma VL was associated with a slight yet statistically significant increase in cardiovascular risk, regardless of CD4 lymphocyte count, ART exposure, and other demographic and clinical factors. These findings contribute to our understanding of the broad spectrum of the VL-cardiovascular risk relationship. In contrast, CD4 lymphocyte count did not demonstrate a connection to cardiovascular risk in any of the adjusted models.

There is currently no universally accepted method for evaluating cardiovascular risk and conveying this risk to youths living with HIV. The conventional approach of expressing increased risk of CVD as a probability of an event over the subsequent 10 years may discourage patients from adhering to healthier lifestyles and preventive care [54]. Using average age as a constant factor (the same for everyone) in our risk scores could pave the way for the development of an EHR-integrated monitoring device that assesses cardiovascular risk in young HIV-positive populations and enables effective risk communication to youths living with HIV. This approach could also have significant implications for clinical decision-making regarding treatment thresholds in youths living with HIV. Since age is a key factor in predicting absolute risk [46], the use of our risk scores could help classify youths living with HIV who are at risk of cardiovascular health issues. Our study provides a foundation for future research that can further investigate this approach and apply it to HIV-negative youth populations. As we obtained similar results from both risk scores, the simpler version could serve as a cost-effective means of assessing high-risk HIV-positive youth for cardiovascular health conditions using readily available clinic-based predictors. However, it is crucial for researchers to periodically assess potential biases that may not have been apparent in this study.

Previous studies have indicated that the FRS is not effective in categorizing lifetime risk in younger individuals [55] and have recommended relative risk estimates instead of age-dependent absolute risk estimates for those with low short-term risk [56]. A study that examined the ability of the FRS and the Adult Treatment Panel III to predict long-term risk for coronary heart disease death in young men aged 18-39 years found that neither method identified individuals under 30 years of age as high risk despite significant risk factor burden [57]. In this study, age was kept constant, meaning all individuals aged 18-29 years were given the risk estimate of a 30-year-old [57]. However, using continuous risk scores in our study could offer advantages over arbitrary classifications of high cardiovascular risk. In a cohort of youths living with HIV with lower event rates than the original Framingham cohort, identifying only high-risk individuals based on a >20% absolute risk in 10 years, despite significant risk factor burden, may not be the most effective strategy for estimating and communicating cardiovascular risk to younger individuals.

The FRS, on the other hand, has its own set of advantages. Given its strengths, we opted to adapt the FRS as a tool to assess CVD risk among youths living with HIV, despite the existence of other risk assessment algorithms. FRS was developed using a large, community-based cohort of individuals from Framingham, Massachusetts [58]. This population was representative of the US population at the time the study was conducted, which is important because HIV-positive individuals in the United States may have different risk profiles compared to those in other regions. FRS is based on traditional CVD risk factors, such as age, gender, blood pressure, total cholesterol, HDL cholesterol, and smoking status [58]. These risk factors are commonly assessed in clinical settings and are readily available in EHRs, making FRS a feasible tool for use in routine clinical care for youths living with HIV. FRS is a simple tool that is easy to use and understand, which may be particularly important for health care providers who may not have extensive training in CVD risk assessment. Other risk assessment algorithms may also be appropriate depending on the specific characteristics of the population being assessed. Nonetheless, there is still a need for further research to establish a definitive and widely accepted standard for assessing the risk of CVD in young adults living with HIV.

Despite the use of a multiclinic sample, there are potential limitations to the current findings. First, the study design was cross-sectional, preventing the establishment of causal inferences. Future research should adopt a longitudinal approach to observe changes over time and test these associations. Second, caution should be exercised when generalizing our findings to the wider population of youths living with HIV in the United States or other locations. For instance, the high proportion of Hispanics in Tampa and a large Black population in Memphis, Tennessee (over 50% of the population), may limit the generalizability of our results to areas with different demographic compositions. Third, we were unable to provide the lower limit detection threshold of VL due to the use of different laboratories and assays across clinical sites, resulting in varied detection limits. Fourth, the use of diagnostic codes as a proxy for substance abuse may have underestimated the prevalence of alcohol and drug use problems. This limitation is further compounded by the high number of missing data, particularly relating to cholesterol, which led to the exclusion of youth from 3 of the highest prevalence areas in the United States. Overall, these limitations suggest that caution should be exercised when interpreting the current findings, and further studies that prioritize more complete EHR data are necessary to address these limitations.

Furthermore, there are several limitations to grouping substances together in our study using EHR data. Combining substances into 1 group may mask potential differences in the cardiovascular effects of individual substances, limiting our ability to identify specific risk factors associated with each substance. This could lead to overgeneralization of findings and inaccurate conclusions about the effects of substance abuse on cardiovascular health. Another limitation of our study is that missing values were assumed as nonsubstance users. This assumption may lead to an underestimation of the prevalence of substance abuse in our sample, as there are issues around documentation and disclosure of alcohol and drug use in clinical settings, which may result in incomplete or inaccurate information in EHR data. This limitation could have implications for the generalizability of our findings, as it may not reflect the true prevalence of substance abuse in the population under study.

Additionally, assuming missing values indicate nonsubstance use could introduce bias if the reasons for missing data are related to substance abuse. For example, individuals who are actively using substances may be less likely to disclose their use or may miss appointments or follow-up visits, leading to missing data. This bias could lead to inaccurate estimates of the prevalence and effects of substance abuse on cardiovascular health. Therefore, while grouping substances and assuming missing values as nonsubstance users may be practical approaches for analyzing EHR data, they do have limitations that need to be considered when interpreting the results of our study.

Despite the limitations, our study is innovative in using EHRs to investigate the independent effects of HIV biomarkers on cardiovascular risk in a US-based cohort of youths living with HIV who received routine medical care. The use of EHRs allowed for the inclusion of HIV outcome data from clinic-based patients, which reduced study costs, and the use of ICD-10 codes to identify clinical covariates for analysis. Our findings have important implications for the development of a multivariable risk assessment method tailored to youths living with HIV. Future research should investigate the inclusion of VL in cardiovascular risk equations for young people to predict CVD risk. Our study emphasizes the importance of recognizing HIV infection as an additional risk factor for CVD and providing preventive CVD care for youths living with HIV in routine practice. Multicomponent interventions that target both VL suppression and cardiovascular risk reduction among youths living with HIV are warranted [25].

Our study also highlights the need for a preventative health life course approach in the care of youths living with HIV. A recently published cohort study found that the risk of CVD remained consistently higher among people with HIV, regardless of age or diagnosis timing [59]. It is important to note that HIV alone, without consideration of other comorbidities and risk factors, may underestimate the burden of CVD among young people living with HIV [1,25]. Therefore, interventions designed with a preventative health life course approach should consider those with particularly elevated cardiovascular risk, especially if risk-enhancing factors related to HIV (eg, low CD4 lymphocyte count or a history of prolonged viremia) are present [60]. Similarly, a syndemics approach is necessary to combat the growing burden of CVD among young adults with HIV.

Syndemics refer to the interaction between 2 or more epidemics that mutually reinforce each other and increase the burden of disease in a population [61]. In the case of youths living with HIV, the interaction between HIV and CVD epidemics is evident, as HIV infection increases the risk of developing CVD, while CVD risk factors are prevalent among individuals with HIV [4,6,25]. Therefore, a comprehensive approach that addresses the interrelated factors contributing to the high burden of CVD in youths living with HIV is necessary. This approach should include interventions that target HIV treatment and management, CVD prevention, and the social determinants of health. By addressing these factors in a coordinated manner, health care providers can reduce the burden of CVD and improve the overall health outcomes of young adults with HIV.

## Appendix

Multimedia Appendix 1 Syntax for Cardiac Risk Score1 and Cardiac Risk Score2.

## Abbreviations

ART: antiretroviral therapy
CD4: cluster of differentiation 4
CONSORT: Consolidated Standards of Reporting Trials
CVD: cardiovascular disease
EHR: electronic health record
FRS: Framingham Risk Score
HDL: high-density lipoprotein
HIPAA: Health Insurance Portability and Accountability Act
ICD-10: International Statistical Classification of Disease and Related Health Problems Tenth Revision
IRB: institutional review board
LS7: American Heart Association’s Life’s Simple 7
VL: viral load

## Data Availability

The corresponding author can provide the data sets that were generated and analyzed during this study upon reasonable request.
