# Supplementary material for: Cardiovascular Risk Assessment Among Adolescents and Youths Living With HIV: Evaluation of Electronic Health Record Findings and Implications
Source: Interact J Med Res. 2023 Aug 16;12:e41574. doi: 10.2196/41574 (PMC10468705; doi:10.2196/41574)
Supplement: Multimedia Appendix 1 [file ijmr_v12i1e41574_app1.docx]

# **Supplementary Materials**

***Cardiac Risk Score1* Syntax**

DO IF Male = 1 AND BP_Meds = 0.

COMPUTE RiskFactors_R1 = ((LN(21))*AgeFactorM) + ((LN(SysBP))*SysBPFactorM)+(Cig*CigFactorM)+(DM*DMFactorM)-AvgRiskM.

END IF.

EXECUTE.

DO IF Male = 0 AND BP_Meds = 0.

COMPUTE RiskFactors_R1 = ((LN(21))*AgeFactorW)+(SysBPFactorW*(LN(SysBP)))+(Cig*CigFactorW)+(DM*DMFactorW)-AvgRiskW.

END IF.

EXECUTE.

DO IF Male = 1 AND BP_Meds = 1.

COMPUTE RiskFactors_R1 = ((LN(21))*AgeFactorM)+ ((LN(SysBP))*SysBPFactorM_med)+(Cig*CigFactorM)+(DM*DMFactorM)-AvgRiskM.

END IF.

EXECUTE.

DO IF Male = 0 AND BP_Meds = 1.

COMPUTE RiskFactors_R1 = ((LN(21))*AgeFactorW)+(SysBPFactorW_med*(LN(SysBP)))+(Cig*CigFactorW)+(DM*DMFactorW)-AvgRiskW.

END IF.

EXECUTE.

DO IF Male = 1.

COMPUTE Risk1 = 100*(1 - RiskPeriodFactorM**EXP(RiskFactors_R1)).

END IF.

DO IF Male = 0.

COMPUTE Risk1 = 100*(1 - RiskPeriodFactorW**EXP(RiskFactors_R1)).

END IF.

EXECUTE.

***Cardiac Risk Score2* Syntax**

DO IF Male = 1 AND BP_Meds = 0.

COMPUTE RiskFactors_R2 = ((LN(21))*AgeFactorM)+(TotalCholFactorM*(LN(TotalChol))) +(HDLCholFactorM*(LN(HDLChol))) + ((LN(SysBP))*SysBPFactorM)+(Cig*CigFactorM)+(DM*DMFactorM)-AvgRiskM.

END IF.

EXECUTE.

DO IF Male = 0 AND BP_Meds = 0.

COMPUTE RiskFactors_R2 = ((LN(21))*AgeFactorW)+(TotalCholFactorW*(LN(TotalChol)) )+(HDLCholFactorW*(LN(HDLChol)) ) + ((LN(SysBP))*SysBPFactorW)+(Cig*CigFactorW)+(DM*DMFactorW)-AvgRiskW.

END IF.

EXECUTE.

DO IF Male = 1 AND BP_Meds = 1.

COMPUTE RiskFactors_R2 = ((LN(21))*AgeFactorM)+ (TotalCholFactorM*(LN(TotalChol))) + ( (LN(HDLChol))*HDLCholFactorM ) + ((LN(SysBP))*SysBPFactorM_med)+(Cig*CigFactorM)+(DM*DMFactorM)-AvgRiskM.

END IF.

EXECUTE.

DO IF Male = 0 AND BP_Meds = 1.

COMPUTE RiskFactors_R2 = ((LN(21))*AgeFactorW)+(TotalCholFactorW*(LN(TotalChol))) + ( (LN(HDLChol))*HDLCholFactorW ) + ((LN(SysBP))*SysBPFactorW_med)+(Cig*CigFactorW)+(DM*DMFactorW)-AvgRiskW.

END IF.

EXECUTE.

DO IF Male = 1.

COMPUTE Risk2 = 100*(1 - RiskPeriodFactorM**EXP(RiskFactors_R2)).

END IF.

DO IF Male = 0.

COMPUTE Risk2 = 100*(1 - RiskPeriodFactorW**EXP(RiskFactors_R2)).

END IF.

EXECUTE.
